# The difficulty grade of laparoscopic hepatectomy for hepatocellular carcinoma correlates with long-term outcomes

**DOI:** 10.1007/s13304-023-01452-4

**Published:** 2023-02-11

**Authors:** Xiaocui Lv, Lina Zhang, Xin Yu, Hong Yu

**Affiliations:** 1 Department of Anesthesia, Sir Run Run Shaw Hospital, Zhejiang University School of Medicine, No. 3 Qingchun East Road, Hangzhou, Zhejiang China; 2 Department of General Surgery, Sir Run Run Shaw Hospital, Zhejiang University School of Medicine, No. 3 Qingchun East Road, Hangzhou, Zhejiang China

**Keywords:** Laparoscopic hepatectomy, Hepatocellular carcinoma, IWATE-difficulties scoring system, Long-term outcomes

## Abstract

The tremendous development of laparoscopic hepatectomy helps to relieve the difficulties encountered during open hepatectomy. Therefore, a difficulty scoring system was produced to assess the difficulty grade of laparoscopic hepatectomy. The aim of this study was to explore whether the IWATE-DSS is comparable to the long-term outcomes of LH for hepatocellular carcinoma. Clinical data from all consecutive patients who underwent laparoscopic hepatectomy for hepatocellular carcinoma at the Sir Run Run Shaw Hospital, Hangzhou, were prospectively collected and reviewed. The difficulty level of the operations was graded using the IWATE-DSS. The perioperative and postoperative outcomes of laparoscopic hepatectomy were compared at each difficulty level. A total of 300 patients underwent laparoscopic hepatectomy for HCC during the study period. The perioperative and postoperative outcomes were significantly different between the groups according to the IWATE-DSS. There were significant differences in both the intraoperative (bleeding control *p* = 0.000; surgical time *p* = 0.000; estimated blood loss *p* = 0.033) and postoperative variables (postoperative hospital stay *p* = 0.005) among these four groups. The 5-year disease-free survival decreased significantly along with the LH difficulty score (*p* = 0.000). The 5-year overall survival also decreased significantly along with the LH difficulty score (*p* = 0.000). IWATE-DSS was significantly correlated with short- and long-term outcomes in patients who underwent laparoscopic hepatectomy for HCC.

## Introduction

Hepatectomy is known as one of the most effective treatments for liver tumors, regardless of the type of tumor. Since 1991, Reich et al. [1] reported the first laparoscopic hepatectomy (LH), and, in China, an increasing amount of attention has been given to its potential for hepatocellular carcinoma (HCC) compared to open resection. Many studies [2–4] have shown that LH has value in terms of postoperative complications and long-term survival for patients who underwent LH for HCC. Therefore, the DSS (difficulties scoring system) is useful for stratifying the difficulty level of LH procedures. Since Ban et al. [5] established the first DSS for LH in 2014, several other DSSs have been established successively. Researchers [6–8] proposed that the IWATE-DSS [9] performed better in predicting the difficulty of LH than the Hasegawa-DSS [10], Halls-DSS [11], and Kawaguchi-DSS [12] according to bleeding control, surgical time, estimated blood loss, postoperative major complications, and postoperative hospital stay. Many studies [13–15] have indicated that these indicators are correlated with perioperative complications. Consequently, we hypothesized that the IWATE-DSS may be a potential way to predict the long-term outcomes of patients who underwent LH for HCC. Hence, the aim of this study was to use a high-volume center’s data to evaluate the impact of LH difficulty on short- and long-term outcomes and to analyze whether the IWATE-DSS was predictive of the recurrence-free and overall survival of patients who underwent LH for HCC.

## Materials and methods

### Study design

We retrospectively investigated all cases of laparoscopic hepatectomy for HCC undertaken between 2001 and 2021 at the Sir Run Run Shaw Hospital. Follow-up data were obtained from our material database and through direct contact via phone with the patients and their families. Finally, 300 patients were evaluated, excluding the patients who could not be reached due to incorrect telephone numbers who were lost to follow-up. Table 1 displays the baseline characteristics of these patients, while Table 2 lists the patients' surgical characteristics and surgical outcomes. The study was approved by the institutional ethics board of Sir Run Run Shaw Hospital of Zhejiang University (NO.: 20210930-31), and the requirement for individual consent for this retrospective analysis was waived.

**Table 1:** Patient demographic characteristic

Variables	Total (*n* = 300)	Group I (*n* = 22)	Group II (*n* = 124)	Group III (*n* = 132)	Group IV (*n* = 22)	*p*
Age, median, (range) year	59 (20–86)	52 (23–81)	57 (20–86)	59 (23–84)	62 (35–86)	0.076
BMI, median, (range), kg/m^2^	22.9 (14.9–36.9)	23.2 (18.4–29.4)	22.8 (15.8–36.9)	22.9 (14.9–30.4)	22.9 (15.6–27.6)	0.913
Resection period (year) (2001–2010 vs. 2011–2021)	83 vs 217	11 vs 11	42 vs 82	25 vs 107	4 vs 18	0.001*
Male gender, *n* (%)	237 (79.0%)	17 (77.3%)	92 (74.2%)	108 (81.8%)	20 (90.9%)	0.174
Hepatitis status (*n*)						
Cirrhosis	116 (39.1%)	8 (36.4%)	42 (33.9%)	55 (41.7%)	11 (50.0%)	0.459
HBV infection	169 (56.3%)	11 (50.0%)	66 (53.2%)	78 (59.1%)	14 (63.4%)	0.738
Liver function						0.021*
Child–Pugh class A	278 (93.0%)	19 (90.9%)	115 (91.9%)	126 (95.5%)	17 (77.3%)	
Child–Pugh class B	21 (7.0%)	1 (4.5%)	9 (7.3%)	6 (4.5%)	5 (22.7%)	
Diabetes mellitus, *n* (%)	30 (10.0%)	2 (9.1%)	15 (12.9%)	12 (9.1%)	1 (4.5%)	0.654
Mean preoperative laboratory results						
Total Bilirubin(μmol/L), median, (ranges)	15.0 (1.7–107.0)	15.0 (4.6–26.1)	13.9 (1.7–62.6)	15.3 (4.2–107.0)	14.95.13–34.5)	0.789
Albumin(g/L)	40.1 (4.8–54.0)	40.5 (35.0–45.9)	40.7 (4.8–51.3)	39.9 (24.9–54.0)	38.3 (27.9–50.2)	0.114
Alpha-fetoprotein(ng/mL), median, (ranges)	5.8 (0–107,583.2)	7.3 (0–12,290.1)	5.8 (0–107,583.2)	5.6 (0–41,612.0)	7.7 (1.6–2042.6)	0.972
Aspartate aminotransferase(U/L), median, (ranges)	30 (11.0–724.0)	27.5 (13.0–81.0)	30.0 (12.0–724.0)	29.5 (11.0–515.0)	36.0 (19.0–250.0)	0.228
Alanine aminotransferase(U/L), median, (ranges)	28 (1.0–565.0)	26 (8.0–73.0)	29 (3–565.0)	25 (1.0–449.0)	32 (13.0–365.0)	0.425
No. of tumors						0.262
Multiple	56 (19.0%)	1 (4.8%)	22 (17.9%)	27 (21.2%)	6 (27.7%)	
Solitary	238 (81.0%)	19 (95.2%)	99 (82.1%)	104 (78.8%)	16 (72.3%)	
Tumor size						0.000*
< 3 cm	120 (40.1%)	13 (59.1%)	65 (52.4%)	42 (31.8%)	0	
≥ 3 cm	173 (59.9%)	7 (31.8%)	57 (46.0%)	87 (65.9%)	22 (100%)	
Proximity to major vessels						0.000*
Present	21 (7.9%)	0	0	15 (11.4%)	6 (27.3%)	
Absent	244 (92.1%)	15 (68.2%)	110 (88.7%)	106 (80.3%)	13 (59.1%)	

*BMI* body mass index, *HBV* Hepatitis B Virus

*p < 0.05 is statistically significant

**Table 2:** Surgical characteristics and surgical outcomes compared with four groups

Variables	Total	Group I	Group II	Group III	Group IV	Univariate
	(*N* = 300)	(*N* = 22)	(*N* = 124)	(*N* = 132)	(*N* = 22)	*p*
Type of resection						0.000*
Hemihepatectomy	53 (17.8%)	0	1 (0.8%)	36 (27.3%)	16 (77.3%)	
Segmentectomy	200 (67.1%)	1 (4.5%)	97 (79.0%)	96 (72.7%)	6 (22.7%)	
Wedge resection	45 (15.1%)	20 (95.5%)	25 (20.2%)	0	0	
Total operation time (min)	160 (40–800)	120 (60–325)	128 (40–380)	179 (50–800)	212 (95–500)	0.000*
Blood transfusion	65 (21.7%)	2 (9.5%)	24 (19.5%)	32 (24.2%)	7 (31.8%)	0.256
Conversion, *n* (%)	33 (11.0%)	2 (9.5%)	10 (8.1%)	19 (14.4%)	2 (9.1%)	0.444
Pringle's maneuver, *n* (%)	68 (25.0%)	0	18 (14.5%)	45 (34.1%)	5 (22.7%)	0.000*
General complications	73 (24.3%)	3 (13.6%)	28 (22.6%)	33 (25.0%)	9 (40.9%)	0.240
Intraoperative blood loss (ml)	200 (5–5000)	200 (50–800)	200 (5–3000)	300 (20–5000)	350 (100–4500)	0.033*
With complications of Clavien–	18 (6.0%)	0	4 (3.2%)	11 (8.3%)	3 (13.6%)	0.084
Dindo grade IIIA or above						
Clavien–Dindo grade						
III	14 (4.7%)	0	3 (2.4%)	8 (6.1%)	3 (13.6%)	
IV	4 (1.3%)	0	1 (0.8%)	3 (2.2%)	0	
V	0	0	0	0	0	
Hospital mortality	6	0	2	4	0	
Hospital stay, day						0.005*
> 9	132 (44.0%)	13 (54.2%)	44 (36.7%)	57 (43.2%)	16 (72.7%)	
≤ 9	168 (56.0%)	8 (33.3%)	79 (65.8%)	65 (49.2%)	6 (27.3%)	

*p < 0.05 is statistically significant

The patients were divided into four groups according to the IWATE-DSS (Table 3): low (difficulty index 0–3), intermediate (difficulty index 4–6), advanced (difficulty index 7–9), and expert (difficulty index 10–12). The criteria of the scoring system were based on tumor location, tumor size, proximity to major vessels, extent of liver resection, liver function, and HALS (hand-assisted laparoscopic surgery)/hybrid [9].

**Table 3:** Parameters and assigned indexes in the IWATE-DSS

IWATE-DSS Parameters	Indexes
Tumor location (Couinaud segment)	
III segment	1
II/VI segment	2
IVb/V segment	3
I/IVa segment	4
VII/VIII segment	5
Tumors size	
< 3 cm	0
≥ 3 cm	1
Proximity to major vessels^a^	
No	0
Yes	1
Extent of liver resection	
Partial resection	0
Left lateral sectionectomy	2
Segmentectomy	3
Sectionectomy and more	4
HALS/Hybrid	
No	0
Yes	− 1
Liver function	
Child Pugh A	0
Child Pugh B	1

^a^Main or second branch of Glisson’s tree, major hepatic vein, or inferior vena cava

### Data collection

The collected data included baseline characteristics (age, sex, ASA, BMI, case number, numbers of tumors, tumor size and location, hepatitis B (HBV), cirrhosis, Child‒Pugh class, DSS classification, preoperative laboratory results), perioperative recordings (total operation time, estimated blood loss, blood transfusion, conversion rate, type of resection, hospital mortality), postoperative data (postoperative complications, length of postoperative hospital stay, hospital mortality), and survival data (overall survival time, disease-free survival time, 3- and 5-year overall survival rates, 3- and 5-year disease-free survival rates). Among these data, preoperative liver function was classified using the Child‒Pugh classification; the tumor location was classified according to Couinaud segmentation; and tumors were classified based on their central locations when they were located in multiple segments or on the junction of two segments [16].

### Surgical procedures

The same experienced surgical team performed all the operations, and all patients underwent multidisciplinary consultations with surgeons, radiologists, sonographers, anesthesiologists, nutritionists, and rehabilitation professionals before the operation. Inflow and outflow control before segmentectomy and hemihepatectomy was routinely performed by the Pringle maneuver, and the majority of the resections were also performed with an intermittent Pringle maneuver.

### Definition of complications

Postoperative complications were defined and classified according to the Clavien‒Dindo classification [17].

### Statistical analysis

Categorical variables are expressed in numerical figures and percentages. Continuous variables were expressed as median values (with the range) and were compared using the Kruskal‒Wallis test. Categorical variables were compared using the Chi-square test or Fischer's exact test when appropriate, and any differences identified were compared using ANOVA. The Kaplan‒Meier method was used to estimate recurrence-free survival (RFS) and overall survival (OS), which were compared using the log-rank test. According to the IWATE difficulty score, all data were analyzed and compared between the four groups.

All statistical analyses were performed with SPSS version 23.0 software (IBM Corporation, Armonk, NY, USA), and statistical significance was accepted at the 0.05 level.

## Results

### Clinical characteristics of patients

The clinical characteristics of the 300 HCC patients are summarized in Table 1, and the patients included 237 (79.0%) males and 63 females (21.0%). A total of 81.0% of all the patients had single tumors; the mean age was 59 years; and the median BMI was 22.9 kg/m^2^. A total of 169 (56.3%) patients had HBV infections, and 116 (39.1%) patients had cirrhosis. Most of the patients (278, 93.0%) were Child‒Pugh class A. Additionally, 200 (67.1%) patients, which were most of them, underwent segmentectomy, and 267 (89.0%) underwent total laparoscopic hepatectomy. Among all the patients, 33 (11.0%) patients had a conversion to open surgery due to large tumors or severe adhesions. Most (72.3%) of the cases occurred from 2011 to 2021.

### Intraoperative outcomes

According to the IWATE-DSS (Table 3), the patients were divided into 4 difficulty groups (Table 2): 22 patients in the low-difficulty group, 124 in the intermediate group, 132 in the advanced group, and 22 in the expert group.

We compared intraoperative outcomes and postoperative complications among the patients classified into the low, intermediate, advanced, and expert groups. The correlation analysis between preoperative factors and difficulty scores showed no differences in age, sex, body mass index, HBV infection, preoperative laboratory results (total bilirubin, albumin, alpha-fetoprotein, aspartate aminotransferase, and alanine aminotransferase) or number of tumors (Table 1).

There were significant differences in the intraoperative variables (bleeding control *p* = 0.000; surgical time *p* = 0.000; estimated blood loss *p* = 0.033) among these four groups. The median operation time was 160 min (range 40–800 min), and the blood transfusion rate was 21.7%. The median blood loss was 200 ml (range 5–5000 ml), and in 25.0% of the surgeries, Pringle's maneuver was adopted to reduce intraoperative blood loss. The operative time and blood loss increased significantly with procedure difficulty (p = 0.000 and p = 0.033, respectively). Conversion to an open procedure was required in 33 (11.0%) patients, with no significant difference noted between the groups (*p* = 0.444), although it reached 14.4% and 9.1% in Group III and Group IV, respectively.

### Postoperative outcomes

The overall postoperative complication rate was 24.3% (*n* = 300 patients) and increased with the DSS difficulty level. This increase was also noted with major postoperative complications (complications of Clavien‒Dindo grade IIIA or above) (*p* = 0.034) and postoperative hospital stay (*p* = 0.005). Postoperative mortality occurred in six patients with liver failure or MODS (multiple organ dysfunction). The median postoperative hospital stay was 9 days.

### Survival outcomes

**Fig. 1:**
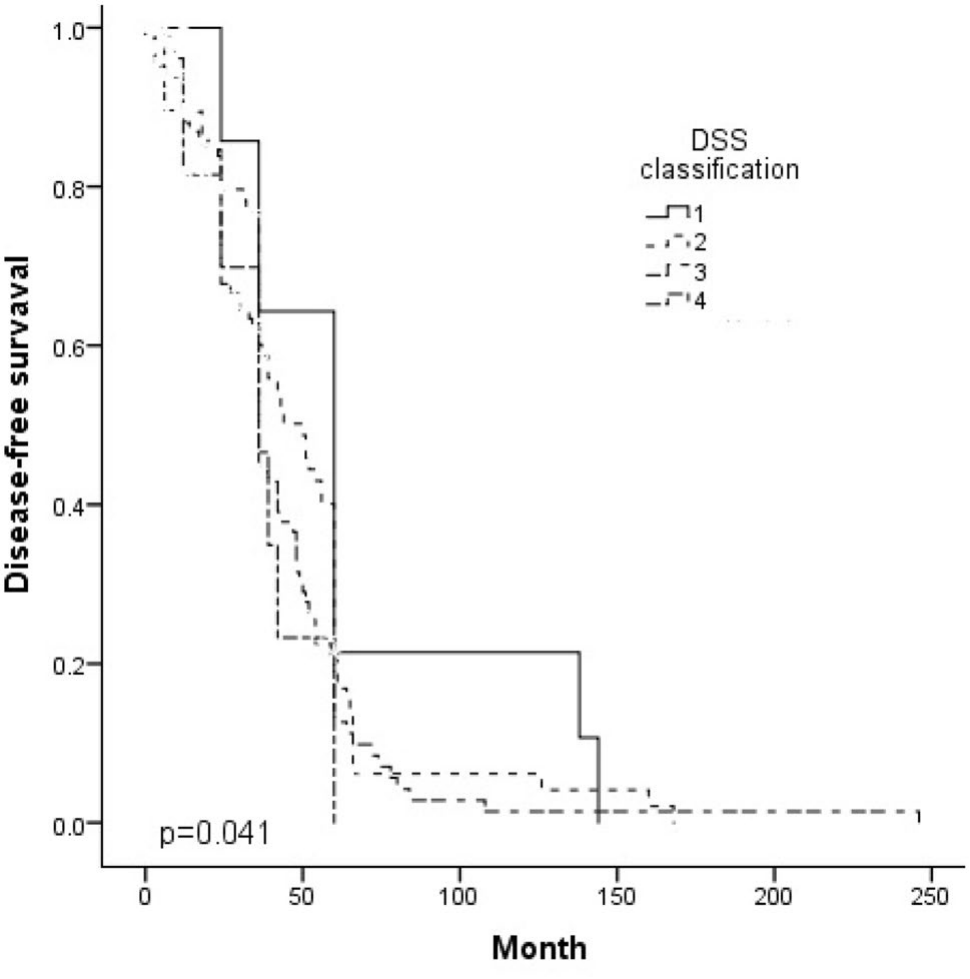
Disease-free survival according to the DSS classification

**Fig. 2:**
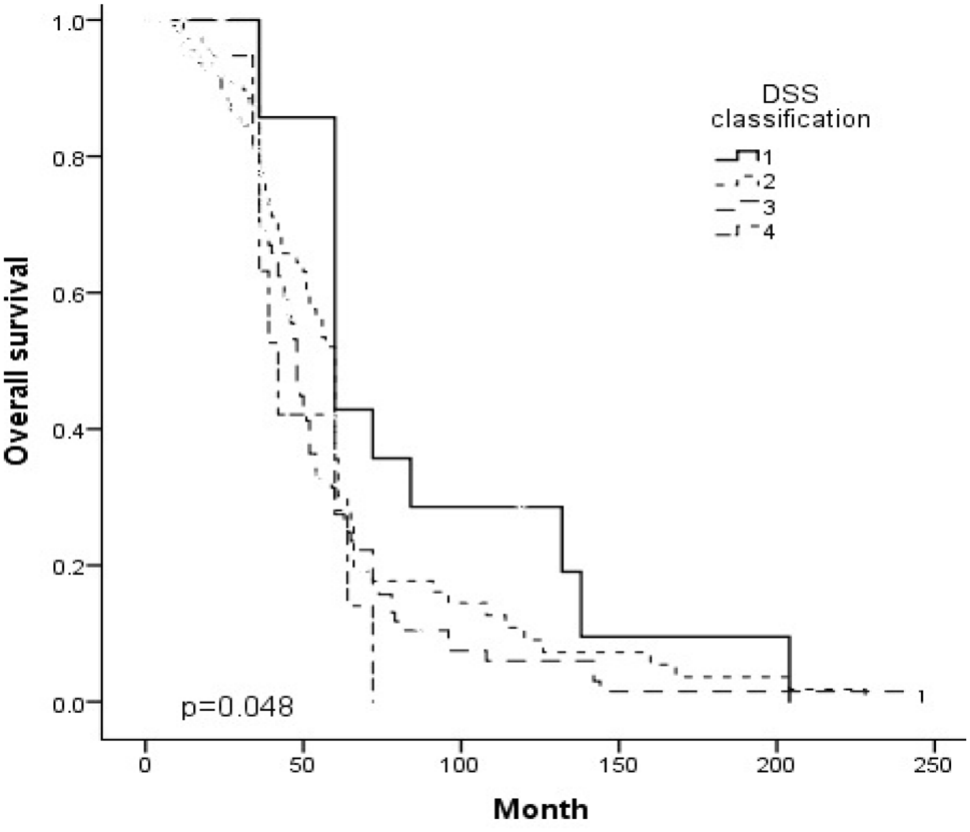
Overall survival according to the DSS classification

The median follow-up time was 36 months (range 0–246 months), and the median disease-free survival was 24 months (0–246 months). The 5-year disease-free survival decreased significantly along with the LH difficulty score (*p* = 0.000). The 5-year overall survival also decreased significantly along with the LH difficulty score (*p* = 0.000). (Table 4) (Figs. 1, 2).

**Table 4:** Oncological outcomes

Variables	Total	Group I	Group II	Group III	Group IV	Univariate
	(*N* = 300)	(*N* = 22)	(*N* = 124)	(*N* = 132)	(*N* = 22)	*p*
Median follow-up, (range), mo	36 (0–246)	60 (6–204)	36 (0–228)	36 (0–246)	24 (3–72)	0.041*
Disease-free survival (DFS)						0.041*
Median DFS, (range), months	24 (0–246)	36 (3–144)	24 (0–168)	24 (0–246)	6 (3–59)	0.033*
3-year DFS, %	43.8	60.0	45.5	42.6	27.3	0.146
5-year DFS, %	18.2	45.0	23.1	11.6	4.5	0.000*
Overall survival, OS						0.047*
Median OS, (rang), months	36 (0–246)	60 (6–204)	36 (0–228)	36 (0–246)	24 (3–59)	
3-year OS, %	57.7	70.0	59.8	57.4	36.4	0.117
5-year OS, %	25.9	60.0	31.1	17.8	13.6	0.000*

*p < 0.05 is statistically significant

## Discussion

This is the first study that describes the relationship between the IWATE scoring system and long-term outcomes of LH for HCC. The current study showed that the IWATE-DSS was significantly correlated with outcome measures associated with intraoperative postoperative and survival outcomes in patients who underwent LH for HCC.

The application of LH has increased exponentially over the past decades. Several researchers [18, 19] have introduced some DSSs to assess the difficulty of LH. The IWATE-DSS, which was a revision of Ban’s first vision, is the most comprehensive to evaluate the difficulty of LH [6–8, 19]. According to the IWATE-DSS, the difficulty of LH is determined by the following factors: tumor size and location, extent of liver resection, proximity to major vessels, liver function, and HALS/Hybrid. Major tumor size [20] and location [21, 22], extent of liver resection [23, 24], and proximity to major vessels [25] tend to increase the difficulty of LH and demonstrate a clear and significant prognostic impact of postoperative results on disease-free survival and overall survival. This single-center retrospective study analyzed the predictive value of the IWATE classification system in a homogenous population consisting of 300 patients who underwent LH for HCC during a period spanning over 20 years.

LH is still a developing field, and the IWATE difficulty scoring system can predict the appropriate surgery well before the operation, and this can serve as a guide determining whether laparoscopic approach can be used. In this study, 300 patients were divided into four groups according to the classification standard: Group I (low), Group II (intermediate), Group III (advanced), and Group IV (expert). Group I did not mean “easy” cases, but the cases were less technically demanding and less complicated than the other groups. The current study showed that there were significant differences in intraoperative bleeding control (*p* = 0.000), total surgical time (*p* = 0.000), and estimated blood loss (*p* = 0.033) among these four groups. All these factors resulted in an increased risk of postoperative complications. The postoperative major complications and the postoperative hospital stay (*p* = 0.005) increased among these four groups. With an increasing classification level, the probability of intraoperative and postoperative complications also increases. The 5-year disease-free survival decreased significantly along with the LH difficulty score (*p* = 0.000). The 5-year overall survival also decreased significantly along with the LH difficulty score (*p* = 0.000). These results demonstrated that the IWATE-DSS can predict long-term outcomes by reflecting intraoperative and postoperative complications during laparoscopic hepatectomy.

Halls[11] confirmed the difficulty scoring system in predicting intraoperative complications during laparoscopic hepatectomy. However, they did not prove the relationship between the difficulty scoring system and long-term outcomes. In our study, we focused on the relationship between the difficulty scoring system and the long-term outcomes. Intraoperative and postoperative complications can definitely affect the overall prognosis and survival time, which has been showed by many researchers [26, 27]. Our results showed that tumor size, type of hepatectomy, liver function, and proximity to major vessels were significantly different among the four groups (Tables 2 and 4). These findings can explain why the IWATE-DSS can fully predict the long-term outcomes in patients who underwent laparoscopic hepatectomy for HCC.

However, the conversion rate was not significantly different. We carefully reviewed all the patient data and found that the vast majority of the patients were diagnosed with HCC through physical examinations, and only a small number of the patients sought treatment after symptoms appeared. This method greatly improves the detection rate of HCC and provides opportunities for the application of laparoscopy, so the proportion of patients in Group IV is lower in the whole dataset (7.3%). An international survey [28] showed that the number of cases of LH were rapidly increasing, and although most of the LH cases were minor LH, the number of major LH cases were also gradually increasing [29]. However, in recent years, ultrasound techniques [30, 31] and Fluorescence Navigation Technology [22] have been introduced into the management of LH, and these can significantly decrease the difficulty of LH and improve prognosis. These two reasons may explain the lack of statistical significance of the conversion rate among the four groups. Barron’s study [7] suggested that the experience gained by surgeons also decreased the conversion rate. This may be another reason. The data from 2011 to 2021 support these findings. Most (72.3%) of the cases occurred in this period (Table 1).

There are still some researchers [32] who believe that the difficulty scoring systems cannot fully assess the difficulty of laparoscopic liver surgery. Several patient factors, such as neoadjuvant chemotherapy, repeated resection, body habitus, BMI, age, and diabetes, can also affect the difficulty of laparoscopic liver resection. In our study, the patients’ data showed that repeated resection (only a small proportion of patients), BMI, age, and diabetes had no influence among the four groups. However, we did not discuss neoadjuvant chemotherapy and body habitus because the time period of our study was 20 years, and some of the patients did not undergo neoadjuvant chemotherapy or use different medicines. Most of China’s HCC cases are caused by HBV infection [33], so we did not include patients’ body habitus. With the changes in Chinese dietary structure and living habits, this may be something we need to consider in the future. For patients who required repeated resection, we found that most of these patients had a longer survival time, which may be because after the first surgery, they needed periodic review. Therefore, the second tumor would be found earlier, and these patients would undergo surgery more aggressively. All the patients had the same surgical team, allowing for consistent medical management throughout the surgical procedure and the patient's hospital stay.

There are some limitations of our study due to its retrospective nature and the single center. First, although we chose all patients who underwent LH for HCC, some data were lost due to the long follow-up time. Selection bias also persisted for economic reasons; some poor patients would not undergo surgery. Second, although all the patients were treated by the same doctor team, the advances in laparoscopic techniques and the experience of the attending physician would have a better impact on the prognosis of subsequent patients. Third, although the scoring system can be performed in the preoperative period, it remains difficult to integrate all risk factors and the objective prediction of the technical difficulty. Last, the long time span of the study may lead to some possible differences between the data obtained early and late in the study.

## Conclusion

We retrospectively analyzed 300 patients’ data in a high-quality hospital and first proposed that the IWATE-DSS significantly correlated with long-term outcomes in patients who underwent laparoscopic hepatectomy for HCC.

## Data Availability

The data generated and/or analyzed during this study are available from the corresponding author on reasonable request.

## Abbreviations

HCC: Hepatocellular carcinoma
LH: Laparoscopic hepatectomy
HBV: Hepatitis B
OS: Overall survival
RFS: Recurrence-free survival

## References

[1] Reich H, McGlynn F, DeCaprio J (1991). Laparoscopic excision of benign liver lesions. Obstet Gynecol.

[2] Deng Z-c, Jiang W-z, Tang X-d (2018). Laparoscopic hepatectomy versus open hepatectomy for hepatocellular carcinoma in 157 patients: a case controlled study with propensity score matching at two Chinese centres. Int J Surg.

[3] Jiang B, Yan X-F, Zhang J-H (2018). Meta-analysis of laparoscopic versus open liver resection for hepatocellular carcinoma. Hepatol Res.

[4] Kobayashi T (2015). Long-term survival analysis of pure laparoscopic versus open hepatectomy for hepatocellular carcinoma in patients with cirrhosis: a single-center experience. Ann Surg.

[5] Ban D, Tanabe M, Ito H (2014). A novel difficulty scoring system for laparoscopic liver resection. J Hepatobil Pancreat Sci.

[6] Lin H, Bai Y, Yin M (2022). External validation of different difficulty scoring systems of laparoscopic liver resection for hepatocellular carcinoma. Surg Endosc.

[7] Barron JO, Orabi D, Moro A (2022). Validation of the IWATE criteria as a laparoscopic liver resection difficulty score in a single North American cohort. Surg Endosc.

[8] Tanaka S, Kawaguchi Y, Kubo S (2019). Validation of index-based IWATE criteria as an improved difficulty scoring system for laparoscopic liver resection. Surgery.

[9] Wakabayashi G (2016). What has changed after the Morioka consensus conference 2014 on laparoscopic liver resection?. Hepatobil Surg Nutr.

[10] Hasegawa Y, Wakabayashi G, Nitta H (2017). A novel model for prediction of pure laparoscopic liver resection surgical difficulty. Surg Endosc.

[11] Halls MC, Berardi G, Cipriani F (2018). Development and validation of a difficulty score to predict intraoperative complications during laparoscopic liver resection. Br J Surg.

[12] Kawaguchi Y, Fuks D, Kokudo N (2018). Difficulty of laparoscopic liver resection: proposal for a new classification. Ann Surg.

[13] Benzoni E, Cojutti A, Lorenzin D (2007). Liver resective surgery: a multivariate analysis of postoperative outcome and complication. Langenbecks Arch Surg.

[14] Katz SC, Shia J, Liau KH (2009). Operative blood loss independently predicts recurrence and survival after resection of hepatocellular carcinoma. Ann Surg.

[15] Lv X, Zhang L, Hong Y, Xin Y (2021). Laparoscopic hepatectomy for hepatocellular carcinoma: short- and long-term outcomes with blood loss. Transl Cancer Res.

[16] Buell J, Cherqui D, Geller D (2009). The international position on laparoscopic liver surgery: the Louisville Statement. Ann Surg.

[17] Dindo D, Demartines N, Clavien PA (2004). Classification of surgical complications: a new proposal with evaluation in a cohort of 6336 patients and results of a survey. Ann Surg.

[18] Russolillo N, Maina C, Fleres F (2020). Comparison and validation of three difficulty scoring systems in laparoscopic liver surgery: a retrospective analysis on 300 cases. Surg Endosc.

[19] Tanaka S, Kawaguchi Y, Kubo S (2019). Validation of index-based IWATE criteria as an improved difficulty scoring system for laparoscopic liver resection. Surgery.

[20] Palanisamy S, Sabnis SC, Patel ND (2015). Laparoscopic major hepatectomy—technique and outcomes. J Gastrointest Surg.

[21] Rotellar F, Pardo F, Martí-Cruchaga P (2017). Liver mobilization and liver hanging for totally laparoscopic right hepatectomy: an easy way to do it. Langenbeck’s Arch Surg.

[22] He J-m, Z-p Zhen Q, Ye,  (2020). Laparoscopic anatomical segment VII resection for hepatocellular carcinoma using the glissonian approach with indocyanine green dye fluorescence. J Gastrointest Surg.

[23] Lee W, Han H-S, Yoon Y-S (2016). Comparison of laparoscopic liver resection for hepatocellular carcinoma located in the poster superiorsegments or anterolateral segments: a case-matched analysis. Surgery.

[24] Pietrasz D, Fuks D, Subar D (2018). Laparoscopic extended liver resection: are postoperative outcomes different?. Surg Endosc.

[25] Gupta R, Fuks D, Bourdeaux C (2017). Impact of intraoperative blood loss on the short-term outcomes of laparoscopic liver resection. Surg Endosc.

[26] Jaeck D, Bachellier P, Oussoultzglou E, Weber JC, Wolf P (2004). Surgical resection of hepatocellular carcinoma. Postoperative outcome and long term results in Europe: an overview. Liver Transplant.

[27] Okamura Y, Takeda S, Fujii T (2011). Prognostic Significance of postoperative complications after hepatectomy for hepatocellular carcinoma. J Surg Oncol.

[28] Hibi T, Cherqui D, Geller DA (2014). International survey on technical aspects of laparoscopic liver resection: a web-based study on the global diffusion of laparoscopic liver surgery prior to the 2nd international consensus conference on laparoscopic liver resection in Iwate. Japan. J Hepatobiliary Pancreat Sci.

[29] Farges O, Goutte N, Dokmak S (2014). How surgical technology translates into practice: the model of laparoscopic liver resections performed in France. Ann Surg.

[30] Ferrero A, Russolillo N, Langella S (2019). Ultrasound liver map technique for laparoscopic liver resections: perioperative outcomes are not impaired by technical complexity. Updates Surg.

[31] Ferrero A, Lo Tesoriere R, Russolillo N (2015). Ultrasound-guided laparoscopic liver resections. Surg Endosc.

[32] Halls MC, Cherqui D, Taylor MA (2018). Are the current difficulty scores for laparoscopic liver surgery telling the whole story? An international survey and recommendations for the future. HPB.

[33] Wang FS, Fan JG, Zhang Z (2014). The global burden of liver disease: the major impact of China. Hepatology.

